# Robust Static Output-Feedback Control for MJLS with Non-Homogeneous Markov Chains: A Comparative Study Considering a Wireless Sensor Network with Time-Varying PER

**DOI:** 10.3390/s21196420

**Published:** 2021-09-26

**Authors:** Arthur R. C. Serafini, Leonardo Delforno, Jonathan M. Palma, Frank H. Behrens, Cecília F. Morais

**Affiliations:** 1 Center of Exact, Environmental and Technological Sciences, Pontifical Catholic University of Campinas, Campinas 13086-900, SP, Brazil; arthur.rcs@puccampinas.edu.br (A.R.C.S.); frank@puc-campinas.edu.br (F.H.B.); 2 Electronics Department, National Service for Industrial Learning (SENAI), Itatiba 13251-360, SP, Brazil; leonardo.delforno@sp.senai.br; 3 Faculty of Engineering, Universidad de Talca, Curico 3340000, Chile; jonathan.palma@utalca.cl

**Keywords:** networked control systems, non-homogeneous Markov chain, wireless network workbench

## Abstract

This paper deals with the problem of control through a semi-reliable communication channel, such as wireless sensor networks (WSN). Particularly, the case investigated is the one where the packet loss rate of the network is time-varying due to, for instance, variation in the distance between the nodes. Considering this practical motivation, the control system is modeled using a formulation based on discrete-time Markov jump linear systems (MJLS) with non-homogeneous Markov chains (time-varying transition probabilities). New control design conditions based on parameter-dependent linear matrix inequalities are proposed in order to solve this problem. The purpose is to demonstrate that this strategy is suitable to handle the networked control problem by comparing the temporal behavior of the closed-loop system with the Markovian controller and a standard proportional-integral-derivative (PID) controller. The case study presented in the paper considers the problem of the remote control of a Vertical Take-Off and Landing (VTOL) vehicle through a wireless communication channel. The network packet loss model employed in the case study is based on data collected on a wireless network workbench, which was previously developed and validated by the authors.

## 1. Introduction

Either for their flexible architecture, or for the low cost of installation and maintenance, networked control systems (NCS) have become more popular in recent decades [1]. The projects based on NCS can be considered multidisciplinary because they represent an intersection between control theory (which considers perfect transmission of measurement and control input, neglecting packet loss, network-induced delays, and uncertain or time-varying sampling rates) and communication theory (which studies the transmission of information by unreliable or semi-reliable channels such as wireless sensor networks—WSN).

The most recent research in the WSN literature is concerned with improving the robustness of the network by using, for instance, topology optimization models to help WSN resist cascading failures [2,3], information fusion processes to lower the negative impact of the environment on message routing [4], and more realistic failure models to analyse the network invulnerability [5]; etc. However, if the network is used as a communication channel for NCS projects, there are some alternative techniques that guarantee the robustness of the closed-loop system even under, for instance, denial of service in cyber-physical attacks [6,7], network-induced delays [8], and packet loss, among several other issues [9,10,11]. In this sense, despite their advantages, the most common and least wanted feature of the NCS is the possibility of packet loss in the network. Packet loss can occur due several factors, such as transmission errors in the physical layer, buffer overflows in networks that are used for distinct purposes and are shared by heterogeneous devices, long transmission intervals that lead to package reordering (old packets are discarded in the control scenario), and so forth.

The first step in designing an NCS that provides theoretical guarantees of closed-loop stability in the presence of the inherent undesirable phenomena, such as packet loss, is to determine the model of transmission error associated with the communication channel. Due to the abrupt changes in its operation point, a suitable modeling for semi-reliable networks can be performed by so-called discrete-time Markov Jump Linear Systems (MJLS) [12]. Those systems are based on a sequence of random variables $\theta_{k}$ (*k* stands for the time) belonging to a finite set of operation modes (called Markov chain) such that the next value of the sequence ($\theta_{k+1}$) depends only on the current value ($\theta_{k}$) and not on the past values. Furthermore, the switching between the modes is governed by a transition probability matrix. Particularly in the NCS context, usually the operation modes are related to successful or failed transmissions.

Most of the papers in MJLS literature are based on a fragile assumption: that the transition probability matrix is time-invariant. However, this hypothesis can be often violated in the NCS scenario because failure transmission probabilities usually depend on external changed environment, age, humidity, lifespan, different transmission rates, distinct communication protocols, distance between nodes, and other factors affecting link signal strength. In this sense, two strategies can be adopted. The first one consists of discretizing the continuously varying probabilities into a limited number of discrete values, creating a class of finite piecewise homogeneous Markov chains [13,14]. The second one, employed in this paper, is to consider a non-homogeneous stochastic process (i.e., the Markov chain is non-ergodic). This technique is appropriate to model, for instance, the problem of control design through WSN when the distance between the source and sink nodes is variable [15], which usually implies the change of the successful transmission probability in the physical layer of the “Open Systems Interconnection” (OSI) model [16]. Some examples that fit in this context involve the control of mobile units such as autonomous vehicles, drones, etc.

Notwithstanding, the time-varying probabilities are a common problem in NCS; most of the research in MJLS control is grounded on the second moment stability (SMS) definition, which encompasses the stochastic stability, mean square stability (MSS), and exponential MSS concepts [17], being valid only for ergodic Markov chains [12] [Assumption 3.31]. Therefore, in this paper, aside from considering the Markovian model of NCS, the non-homogeneous Markov chain (time-varying probabilities) is also considered, aiming at avoiding instability or performance degradation caused by the misuse of controller designs based on classical stability concepts (such as proportional-integral-derivative—PID) [18,19,20] or on SMS-based techniques.

The main contribution of this paper is the new control strategy (proposed in Theorem 1 and Corollary 1 in Section 5) that are based on the stochastic stability analysis results of [21] for discrete-time MJLS with a non-homogeneous Markov chain. The controller synthesis is performed by means of parameter-dependent linear matrix inequality (LMI) conditions for state-feedback control of this class of system. Nevertheless, in order to compare the time-behavior of the closed-loop networked control system output using a standard PID controller versus a Markovian one, the LMI conditions from Theorem 1 are adapted to provide a robust (parameter-independent) controller. The robustness against the variation of the probabilities is achieved by fixing the slack variables used to recover the gain. One novelty when compared with other control literature research on non-homogeneous MJLS is that this paper handles static output-feedback (SOF) control (which is usually a non-convex problem, but more applicable in practice), while previous works [21,22,23,24] investigate state-feedback (SF) stabilization or stability analysis. The adaptation from SF to SOF is assured by Assumption 1 in Section 4, imposing a mask to the gain, that is, a particular structure to the slack variables in order to select only the system states available for feedback. Finally, the case study presented at the end of the paper is motivated by a practical problem of SOF control of a Vertical Take-Off and Landing (VTOL) helicopter with remote sensor and remote actuator. To increase the reliability of the numerical experiment, an additional novelty in relation to previous MJLS control research consists of using the data provided by a wireless network workbench [25] to compute, with good accuracy, a Markovian model for the communication channel used in the case study. Despite the fact that mathematical models for the signal attenuation as a function of time and distance can be found in the WSN literature, another motivation for using experimental information representing the packet loss on WSN is to provide data for control designers without the need for them to be familiar with the communication theory.

This paper is organized as follows. Section 2 presents some related works, discussing WSN performance analysis tools and their application to NCS problems. Section 3 contains a detailed description of the wireless network workbench that provides the necessary data to compute the Markovian model for the communication channel employed in the case study. Section 4 presents the MJLS model and the mathematical formulation of the NCS problem. Section 5 develops the theorem for the design of non-homogeneous Markov control. Section 6 presents a case study of a VTOL vehicle remote control through WSN with the purpose of comparing the performance of PID and Markov controllers. Finally, Section 7 presents the final considerations and proposals for future works.

**Notation**: Capital and lowercase letters are used to, respectively, represent matrices and vectors (or scalars). The set of real numbers is denoted by R. For real matrices and vectors the transposition is represented by the symbol ${(}^{\prime })$, while (⋆) is used for blocks induced by symmetry in square matrices. For symmetric matrices P>0 or P<0, respectively, denote that *P* is positive or negative definitely. $\cup_{f\in F}M_{f}$ and $\cap_{f\in F}M_{f}$, respectively, stand for the union and intersection of sets $M_{f}$ where the index *f* belongs to the set *F*. Diag(·) represents a block-diagonal matrix.

## 2. Related Works

Analytical modeling methods for WSNs require some simplifications to predict their performance. Thus, they become inadequate in certain analyses due to the inherent complexity and diverse nature of WSNs (e.g., node density, node mobility, dynamic topology, wireless media characteristics, time-varying channel conditions, etc.). Oversimplified models may lead to inaccurate results that are not desirable. On the other hand, it may not be feasible or practical to test and evaluate the performance of protocols through their actual implementation because this is a complex, expensive and time-consuming activity. In this scenario, several works in the literature (see [26,27,28,29,30,31,32] and references therein) propose analyzing tools for evaluating algorithms and protocols on WSNs at the design, development, and implementation stages, such as simulators, emulators, and testbeds. Each one of those tools has different features, characteristics, models and architectures for performance testing on WSNs. Briefly, such tools can be described as follows: simulators are based on software that models the real environment; emulators differ from simulators because they can run the same code on real platforms; finally, physical testbeds allow more detailed real-world settings and assist in capturing realistic experimental data.

In the early design and development phase, the use of simulators can be considered a good choice because they provide a higher level of abstraction. For example, the design and development of protocols for routing and topology control decisions can be made based on the simulation. Simulation-based approaches provide some advantages, such as lower costs, scalability, shorter test execution times, and ease of implementation. Furthermore, time, effort, and resources required for the simulation are minimal [29,30].

Emulation is a hybrid approach that combines hardware and software in which some components are implemented in real hardware (e.g., sensor nodes) and some are simulated (e.g., links, traffic etc.). Emulators are more effective and useful, especially when testbeds are not available or cannot be deployed due to the characteristics of the applications. Since emulators run the same code in real nodes, they reduce the implementation effort, and the results obtained are more accurate than simulators [29,30].

Although simulators and emulators are valuable tools for evaluating the performance of WSNs, unrealistic assumptions and simplified models lead to inaccurate results. To solve this problem, the testbeds bridge the gap between simulation and actual deployment. They provide an environment for protocol testing and evaluation similar to real deployment and offer the opportunity to configure, run, and monitor experiments remotely while evaluating models, algorithms, protocols, and applications. The results obtained through tests are more accurate than with software-based tools [31,32].

The research reported in [26] presents a survey of thirty-five assessment tools for WSN performance that were available in 2010. That paper performs a horizontal and vertical analysis of these tools, which were selected based on their popularity, support availability, active maintenance, and available help. In the horizontal dimension, outstanding competitors were selected at each stage of WSN development. For vertical analysis, simulation, emulation, and testbeds tools were categorized according to their applicability at each stage. The authors of [26] reinforce that analytic modeling, simulation, emulation, testbed, and real deployment are the most common techniques used for performance analysis of WSNs.

Particularly, [27] presents a deeper review of concepts, characteristics, and limitations of alternatives for simulators, emulators, and testbeds, presenting twenty-three simulators and emulators in detail. On the other hand, [28] focuses specifically on testbeds, analyzing sixteen testbeds, some of them also reported in [26], but extending the analysis to some new ones developed between 2010 and 2016. The authors conclude that testbeds represent WSNs more accurately, support the great diversity components of hardware and software, and allow the deployment of a WSN under the same environmental conditions as a real application. Testbeds can reveal WSN behavior under the effect of failures and malfunctions, a situation that cannot be analyzed in theoretical simulations. A malfunction can occur due to failing hardware, buggy software, power outages in battery-operated systems, and interference with radio communication. In short, testbeds are the ideal environment to find solutions for the occurrences of failures and malfunctions and to assess the interference from the environment. However, [28] states that testbeds are often inadequate for experimentation scenarios that require the repeatability of experiments, since many relevant operational parameters are beyond the user’s control, such as local radio interference due to infrastructure and other experiments.

For the reasons listed above, it is considered that emulation workbenches are a good compromise between simulation and testbed, since they reproduce some characteristics intrinsic to hardware problems but guarantee greater repeatability for the production of statistical data. Note that a wireless network workbench may be a suitable option to obtain probabilistic data for NCS design, since the employment of closed mathematical models [3,4] or those based on algorithms [2,5] requires more theoretical knowledge. On the other hand, open simulators such as *ns-3* [33] or proprietary simulators such as Scalable-Networks https://www.scalable-networks.com/ accessed on 11 September 2021 (specialized solutions) also require prior knowledge.

The laboratory emulation of communication systems for use in the transmission of control signals can also be carried out by means of, for instance, specialty hardware (as supplied by https://www.opal-rt.com/ accessed on 11 September 2021 Opal-RT [34], which is based on cyber-attack modeling in digital communication), with all the characteristics that a closed platform implies. Otherwise, the alternative is the use of simple hardware, as performed in [35], involving only measuring a small set of parameters in the network. However, since each element of the workbench described in Section 3 is modular, the radio frequency devices can be changed for the study of such commercial communication standards as Wi-Fi, Zigbee, Bluetooth, GSM/GPRS, and LoRaWAN, as well as expansion for other future NCS investigations.

## 3. Wireless Network Workbench

As mentioned in the previous section, the state-of-the-art of Wireless Network is composed of a vast literature and frameworks regarding: mathematical modeling of the communication channel [36], simulation [26,27,29,30], emulation, and testbeds [26,27,28,31,32], developed to help analyze the effect of digital networks under actual scenarios, considering channel inspections (physical layer), congestion, collision, and packetization (link and transport layer). Although the literature is extensive, the use of those tools requires specific knowledge. As an example, one can cite the discrete-event network simulator for Internet systems, ns-3 [33], a free, open-source software whose data collection can be hampered and possibly lead to misinterpretation if users are not particularly familiar with the framework.

Concerning modeling and control in engineering, in which the designers must guarantee the stability of closed-loop systems and, usually, the elements (controllers, actuators, sensors) employ a point–point link, it would be useful to simply obtain the network parameters by means of a user-friendly platform. In this sense, the wireless network workbench considered in this work was developed by two of the authors, and a more detailed description can be found (in Brazilian Portuguese) in [25], which allows the users to evaluate and test protocol stacks, attenuation behavior, data rates, system losses, transmission power setups, modulations, carrier frequency, and channel spacing, among other things, for point-to-point communication, and also generates the statistical data that can be employed in NCS design. Despite propagation models being well established in communication theory literature, for didactic purposes, the workbench can be useful to validate the signal propagation models, allowing comparisons between measured and calculated results, and providing data for NCS and the cyber-physical systems research community.

**Figure 1:**
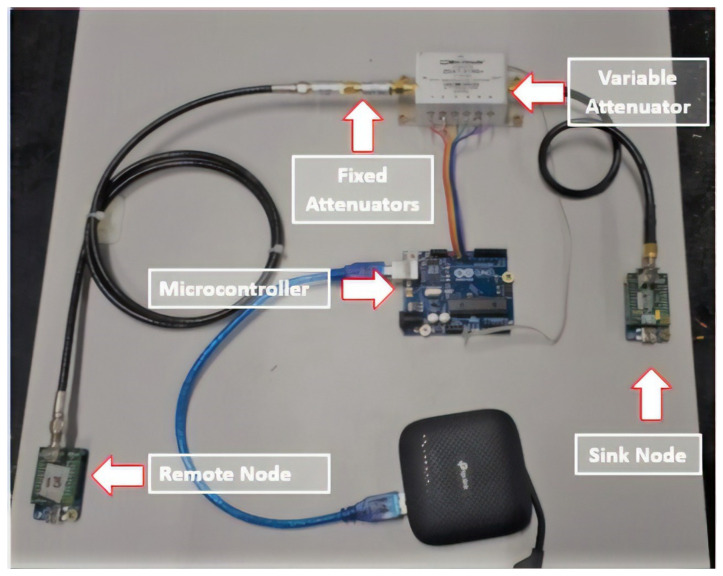
Components of the workbench assembly.

The current setup of the workbench enables us to emulate the behavior of a two-node low-power network under different operating conditions, as shown in Figure 1.

The open-source nature of this workbench ensures full control over the firmware, software, and hardware. In this sense, despite allowing the use of any radio communication solutions, the current configuration of the workbench employs the Radiuino Platform [37], composed of a pair of radio communication modules, BE900 operating at the Industrial, Scientific, and Medical (ISM) frequency of 915 MHz.

**Figure 2:**
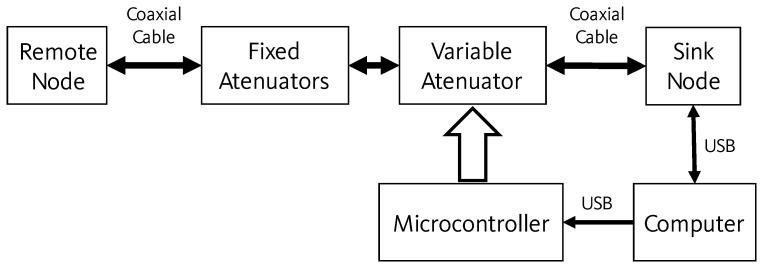
The workbench components and connection setup.

As shown in Figure 2, the workbench requires a personal computer (PC) to control the emulation process, which is connected to the sink node and to a microcontroller via Universal Serial Bus (USB) cables. A variable attenuator and a set of fixed attenuators lie between the sink and the remote nodes, connected to both via coaxial cables, replacing the antennas to ensure a confined and ideal propagation path of the radio frequency (RF) signal with no external interference. The microcontroller module controls the attenuation level in the link between sink and remote nodes.

The deterministic constants of the network are reproduced by the fixed attenuators and the systemic losses. These can be modified by the addition or subtraction of fixed attenuators, while the variable attenuator, ranging from 0 to 31.5 dB, characterizes the environmental conditions.

The emulation software, written in Python (Annexes A and B in [25]), runs in the PC and controls both the sending of communication packets between the sink and the remote nodes, and the communication path constraints. The PC generates the attenuation values that are sent to the microcontroller which, in turn, sets the attenuation level on the variable attenuator, so emulating the communication channel.

For each packet sent from the sink node to the remote node, a new attenuation value is set in the digital attenuator, aiming to emulate the signal propagation behavior in a variety of conditions. The remote node then measures and returns the Received Signal Strength Indicator (RSSI) to the sink node.

The attenuation levels sent to the variable attenuator are defined by the signal propagation models included in the emulation software. Three model options are available in [36] [Chapter 3]: Free Space, Log-distance and Shadowing models. While the first two models consider only deterministic parameters depending on environmental conditions, the Shadowing model adds a log-normal variable whose standard deviation characterizes the environment. The uncertainty introduced by this variable can cause unpredictability in the data transfer rate.

As described in [36] [Chapter 3, Section 3.9.1], it is observed that in the theoretical models and in those based on experimental measurements, the average power of the received signal decreases logarithmically with distance. The average path loss can be expressed as a function of exponent *n*, according to Equation (1).

(1) $$\bar{PL}\left(d\right)\propto {\left(\frac{d}{d_{0}}\right)}^{n}$$

The log-distance path loss model is defined by [36] [Chapter 3, Section 3.9.1]

(2) $$\bar{PL}\left(d\right)\left[dB\right]=\bar{PL}\left(d_{0}\right)\left[dB\right]+10n\log\left(\frac{d}{d_{0}}\right)$$

where *n* is the path loss exponent that describes the rate of loss increase in relation to the reference distance of measurements near the transmitter $d_{0}$. The value of *n* depends on the propagation environment (n=2 for free space and n>2 for environments with obstructions, see Table 1). $\bar{PL}\left(d\right)$ is the average path loss calculated over distance *d*.

The model of Equation (2) does not consider the surrounding environmental clutter, which can interfere in a distinct way in two different places at the same distance *d*. Experimental data demonstrate that the path loss PL(d) in a particular position is random and log-normally distributed (normal distribution in dB).

The log-normal shadowing model adjusts Equation (2) to take into account statistically distributed variations experimentally observed: [36] [Chapter 3, Section 3.9.2]

(3) $$PL\left(d\right)\left[dB\right]=\bar{PL}\left(d_{0}\right)\left[dB\right]+10n\log\left(\frac{d}{d_{0}}\right)+XdB\left[dB\right]$$

where XdB is a random variable with zero mean and standard deviation σ expressed in dB, and it is associated with probabilistic models that determine the characteristics of the environment. Finally, based on Equation (3), it is possible to determine the power received at distance *d* ($P_{r}\left(d\right)$) as a function of the transmitted power ($P_{t}$) and path loss (PL):

(4) $$P_{r}\left(d\right)\left[dB\right]=P_{t}\left[dB\right]-PL\left(d\right)\left[dB\right]=P_{t}\left[dB\right]-\left(10n\log\left(\frac{d}{d_{0}}\right)+XdB\left[dB\right]\right)$$

The use of probabilistic models is useful to describe the unguided propagation of signals. A continuous random variable can take an infinite number of possible values. This feature can be used in radio signal modeling. In probability theory, the normal distribution (or Gaussian distribution) is a continuous probability distribution, used for fluctuations around a mean value μ and standard deviation σ. The log-normal random variable XdB is closely related to environmental conditions with the standard deviation $\sigma_{dB}$, highlighting the types of environment as shown in Table 2 [38].

**Figure 3:**
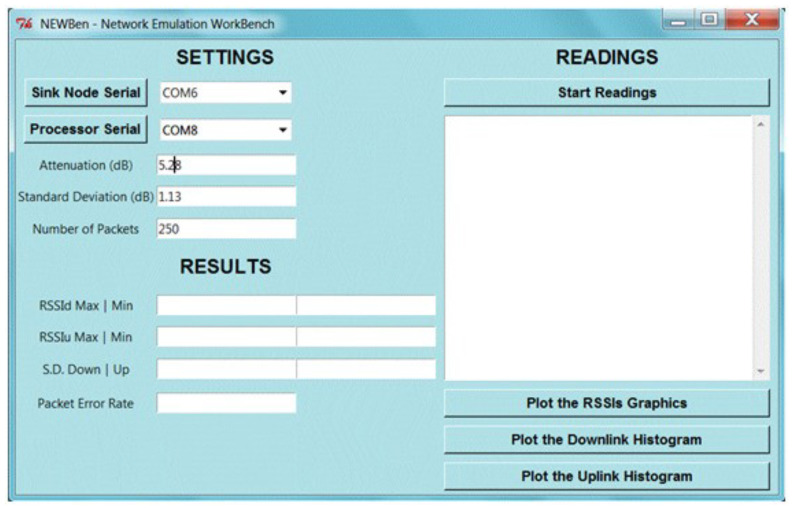
The HMI showing the settings and results menus.

Before launching the emulation software, the Human–Machine-Interface (HMI) shown in Figure 3 needs to be fulfilled with the data from the network to be emulated, and the serial ports of the microprocessor and sink node must be chosen.

As the emulation progresses, the interface presents the number of the current packet and corresponding down and uplink RSSI. After the transmission is complete, the software generates reports that include the following information: the Packet Error Rate (PER), the maximum, minimum and mean RSSI, and the standard deviation of the RSSI.

The proposed wireless network workbench was validated by comparing its output data with data obtained in experimental field tests. In this sense, a series of tests were conducted to compare the signal behavior obtained from the workbench against actual, point-to-point, reference test networks, using the same communication modules and configuration parameters, that is, 10 dBm transmission power, 2FSK modulation technique, 9600 bps baud rate, 38.38 kbps data rate, and 125 kHz channel spacing.

Table 3 shows that the emulation results obtained by the workbench are very close to the ones provided by the experimental field tests in terms of signal intensity, mean and standard deviation, attesting the quality of the emulation offered by the workbench.

To analyze the WSN packet loss behavior as the distance between nodes increases, two additional tests were performed considering a fixed standard deviation in 1.13, but choosing two opposite extremes for the signal attenuation values: 1 dB representing a minimum distance between the nodes and 25 dB representing the two nodes 310 m apart. Table 4 shows the results for these tests.

It is possible to notice an increase in the packet loss as the distance between nodes increases, that is, the PER is 11% for the longest distance (310 m), while for the minimum distance it reduces to 1%.

### 3.1. Markovian Model for the Communication Channel

Aside from PER, maximum, minimum, mean and standard deviation of RSSI, the wireless network workbench provides a report where the sequence of sent and received packets can be found. Since the emulated network is a semi-reliable communication channel, the sink node may indicate some packet loss; that is, each packet sent by the source node can be associated with a successful ($\theta_{k}=1$) or failure transmission ($\theta_{k}=2$). For instance, consider the case illustrated in Table 5 supposing that only 10 packets (numbered from 1 to 10) are sent by the source node, and the report provided by the workbench indicates that packets 2 and 5 were not received. Note that $\theta_{k}$ represents a random variable at discrete-time instant *k* that assumes values on a finite set K={1,2,…,η} containing all the η operation modes of the system or all the η states of a Markov chain. In this particular example, one has only two states, that is, K={1,2}, where the first one corresponds to success and the second one represents failure in the transmission.

**Figure 4:**
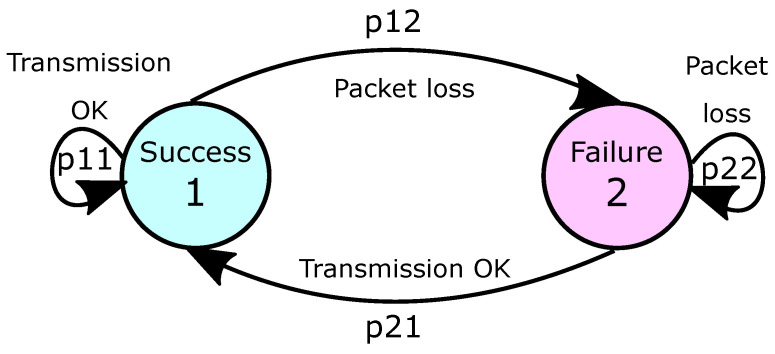
Markov chain for the Gilbert-Elliot model.

From Table 5, four different transition states can be identified: $t_{11}$ (two consecutive successful transmissions), $t_{12}$ (a successful transmission followed by a failed one), $t_{21}$ (a failed transmission followed by a successful one), and $t_{22}$ (two consecutive failed transmissions, which do not occur in this particular example). As a consequence, the packet loss on the communication channel can be modeled by the so-called Gilbert-Elliot model [39,40,41], whose transitions among the states are described by the diagram of Figure 4.

Assuming a Gilbert-Elliot model, the transition probabilities among states $\theta_{k}=1$ (Success) and $\theta_{k}=2$ (Failure) are given by

$$\begin{array}{c} p_{11}=\mathrm{Prob}\left(\theta_{k+1}=1|\theta_{k}=1\right)=\frac{t_{11}}{t_{11}+t_{12}},\;p_{12}=\mathrm{Prob}\left(\theta_{k+1}=2|\theta_{k}=1\right)=\frac{t_{12}}{t_{11}+t_{12}},\\ p_{21}=\mathrm{Prob}\left(\theta_{k+1}=1|\theta_{k}=2\right)=\frac{t_{21}}{t_{21}+t_{22}},\;p_{22}=\mathrm{Prob}\left(\theta_{k+1}=2|\theta_{k}=2\right)=\frac{t_{22}}{t_{21}+t_{22}}. \end{array}$$

For a robust set of data that can be provided by the workbench, the time-evolution of the random variable $\theta_{k}$ can be represented with good accuracy by a Markov chain associated with a transition probability matrix $P=[p_{ij}]$, where $p_{ij}=\mathrm{Prob}\left(\theta_{k+1}=j|\theta_{k}=i\right)$, $p_{ij}\geq 0$, ∀i,j∈K={1,η} and $\sum_{j=1}^{\eta }p_{ij}=1$, ∀k≥0. In the NCS context, the transition probability matrix governs the jumps among the η operation modes and, consequently, the system dynamics behavior. For a Gilbert-Elliot model, η=2 and

$$P=\left[\begin{array}{cc} p_{11} & p_{12}\\ p_{21} & p_{22} \end{array}\right]=\left[\begin{array}{cc} 1-p_{12} & p_{12}\\ 1-p_{22} & p_{22} \end{array}\right].$$

The statistical information provided by the workbench result in the following probability matrix is

(5) $$P\left(\alpha \right)=\left[\begin{array}{cc} 1-PER(\alpha ) & PER(\alpha )\\ 0.999 & 0.001 \end{array}\right].$$

Note that the packet error rate (PER) provided on the workbench’s HMI (Figure 3) actually has a direct relationship with the adopted Markovian model. Since the PER values can change for distinct reasons, such as bandwidth (data rate) or attenuation levels, it is considered that PER and, consequently, P depend on parameter α; more details about this parameter are presented in the next sections.

## 4. Discrete-Time Markov Jump Linear System

Knowing that the Markov jump linear system (MJLS) class can model systems that suffer abrupt changes in their operation point, such as packet loss in NCS, consider a discrete-time MJLS represented by

(6) $$G=\left\{\begin{array}{cc} x(k+1) & =A\left(\theta_{k}\right)x\left(k\right)+B\left(\theta_{k}\right)u\left(k\right),\\ y(k) & =C\left(\theta_{k}\right)x\left(k\right) \end{array}\right.$$

where $x\left(k\right)\in R^{n_{x}}$ is the state vector, $u\left(k\right)\in R^{n_{u}}$ is the control input vector, $y\left(k\right)\in R^{n_{y}}$ represents the measured outputs, and $\theta_{k}$ is a random variable that assumes values on a finite set K={1,2…,η} that represents η linear operation modes of the MJLS. As described in Section 3.1, the transition among subsystems depends on the associated Markov chain, with the transition probability matrix given by $P\left(k\right)=[p_{ij}\left(k\right)]$, where $p_{ij}\left(k\right)=\mathrm{Prob}\left(\theta_{k+1}=j|\theta_{k}=i\right)$, $p_{ij}\left(k\right)\geq 0,\forall i,j\in K$ and $\sum_{j=1}^{\eta }p_{ij}\left(k\right)=1$, ∀k≥0. If the transition probability matrix is time-invariant (P(k)=P, ∀k≥0), the Markov chain is homogeneous, otherwise, it is non-homogeneous [42]. To ease the notation, whenever $\theta_{k}=i$, one writes $A\left(\theta_{k}\right)=A_{i}$, $B\left(\theta_{k}\right)=B_{i}$, and $C\left(\theta_{k}\right)=C_{i}$, ∀i∈K.

In this paper, the non-homogeneous case is considered. A more generic representation of the time-varying transition probability matrix can be found in [24], where, besides the polytopic model, it considers that each entry $p_{ij}\left(k\right)$ can belong to a known interval, or is unknown ($p_{ij}\left(k\right)=?$). Thus, the entries $p_{ij}\left(k\right)$ of the transition probability matrix are not constant, and P(k) can be represented in terms of a polytope (convex combination of *N* known vertices

(7) $$P\left(k\right)=\sum_{m=1}^{N}\alpha_{m}\left(k\right)P_{m},\;\alpha \left(k\right)\in \Lambda$$

where $\alpha \left(k\right)={\left[\alpha_{1}\left(k\right),\ldots ,\alpha_{N}\left(k\right)\right]}^{\prime }$ is a time-varying parameter vector that lies in the unit simplex, ∀k≥0

$$\Lambda =\left\{\alpha \in R^{N}:\sum_{m=1}^{N}\alpha_{m}=1,\;\alpha_{m}\geq 0,\;m=1,\ldots ,N\right\}.$$

Assuming a mode-dependent static output-feedback control law robust to the time-varying parameters

(8) $$u\left(k\right)=L\left(\theta_{k}\right)y\left(k\right)$$

For system (6), the resulting discrete-time closed-loop MJLS is given by

(9) $$x\left(k+1\right)=A_{cl}\left(\theta_{k}\right)x\left(k\right)$$

where $A_{cl}\left(\theta_{k}\right)=A\left(\theta_{k}\right)+B\left(\theta_{k}\right)L\left(\theta_{k}\right)C\left(\theta_{k}\right)$.

**Assumption** **1.** *For the particular case where the output matrix $C(\theta_{k})$ is composed by unit vectors $e_{i}\in R^{n_{x}}$, where $e_{i}$ is the i-th row of an identity matrix with $n_{x}$ rows and columns, one has that the product $L\left(\theta_{k}\right)C\left(\theta_{k}\right)$ can be replaced by a sparse matrix $K\left(\theta_{k}\right)\in R^{n_{u}\times n_{x}}$ with some null entries. For example, suppose $n_{x}=4$, $n_{u}=n_{y}=2$ and $C\left(\theta_{k}\right)={\left[e_{2},e_{4}\right]}^{\prime }$, then*

(10) $$\begin{array}{cc} K(\theta_{k}) & =L\left(\theta_{k}\right)C\left(\theta_{k}\right)=\left[\begin{array}{cc} \ell_{11} & \ell_{12}\\ \ell_{21} & \ell_{22} \end{array}\right]\left[\begin{array}{cccc} 0 & 1 & 0 & 0\\ 0 & 0 & 0 & 1 \end{array}\right]\\ & =\left[\begin{array}{cccc} 0 & \ell_{11} & 0 & \ell_{12}\\ 0 & \ell_{21} & 0 & \ell_{22} \end{array}\right] \end{array}$$

*This means that the problem of designing a static output-feedback controller, described in the next section, is formulated in terms of the synthesis of a state-feedback (SF) control law with the structure constraint*

(11) $$u\left(k\right)=K\left(\theta_{k}\right)x\left(k\right).$$

Furthermore, despite the SOF and SF control laws presented in (8) and (11), respectively, being mode dependent, the design conditions proposed in the next section consider that the information about η operation modes of the Markov chain cannot be fully available. This means that there exist $\eta_{c}\leq \eta$ disjoint groups (clusters), whose union generates the set K, i.e., $K=\cup_{q\in Q}U_{q}$ such that $\cap_{q\in Q}U_{q}$ with indexes $q\in Q=\{1,2,\ldots ,\eta_{c}\}$.

Finally, aiming to assure the stochasticFinally, aiming to assure the stochastic stability of a closed-loop non-homogeneous MJLS, this paper adopts the concept of exponential stability in the mean square sense with conditioning of type I (ESMS-CI) presented in [21,22] and whose further details can be found in [43] [Page 68 Definition 3.1 (*c*)]. Therefore, before presenting the control synthesis conditions, consider that the stability of MJLS (6) with u(k)≡0 and polytopic transition probability matrix P(k) affected by arbitrarily fast time-varying parameters is guaranteed by the next lemma (which is an extension of Proposition 1 from [22]).

**Lemma** **1.** *MJLS (6) with u(k)≡0 is ESMS-CI if, and only if, there exist $P_{i}\left(\alpha \left(k\right)\right)=P_{i}\left(\alpha \left(k\right)\right)>0$ such that the parameter-dependent inequalities*

(12) $$A_{i}^{\prime }P_{pi}\left(\alpha \left(k+1\right)\right)A_{i}-P_{i}\left(\alpha \left(k\right)\right)<0$$

*with $P_{pi}\left(\alpha \left(k\right)\right)=\sum_{j=1}^{\eta }p_{ij}\left(k\right)P_{j}\left(\alpha \left(k\right)\right)$, hold for each i∈K and for all α(k)∈Λ, ∀k≥0.*

## 5. Control Problem in Semi-Reliable Network

This section addresses the problem of designing a controller for a system whose communication with the plant is performed through a semi-reliable network. The following theorem presents sufficient parameter-dependent LMI conditions for state-feedback control of the non-homogeneous MJLS (6).

**Theorem** **1.** *If there exist symmetric positive definite parameter-dependent matrices $W_{i}\left(\alpha \left(k\right)\right)\in R^{n_{x}\times n_{x}}$, ∀i∈K, matrices $H_{\ell }\in R^{n_{x}\times n_{x}}$ and $Z_{\ell }\in R^{n_{u}\times n_{x}}$, ∀ℓ∈Q, such that*

(13) $$\left[\begin{array}{cc} W_{i}\left(\alpha \left(k\right)\right)-H_{\ell }-H_{\ell }^{\prime } & ⋆\\ \Upsilon_{i}{\left(\alpha \left(k\right)\right)}^{\prime }\left(A_{i}H_{\ell }+B_{i}Z_{\ell }\right)\; & -Diag\left(\Upsilon_{i}\left(\alpha \left(k\right)\right)\right)W\left(\alpha \left(k+1\right)\right) \end{array}\right]<0,$$

*holds for all α(k)∈Λ, ℓ∈Q and i∈K, with*

$$\begin{array}{cc} \Upsilon_{i}\left(\alpha \left(k\right)\right) & =\left[\begin{array}{ccc} p_{i1}\left(\alpha \left(k\right)\right)I_{n_{x}} & \cdots & p_{i\eta }\left(\alpha \left(k\right)\right)I_{n_{x}} \end{array}\right],\\ W(\alpha (k+1)) & =Diag\left(\begin{array}{c} W_{1}\left(\alpha \left(k+1\right)\right),\cdots ,W_{\eta }\left(\alpha \left(k+1\right)\right) \end{array}\right) \end{array}$$

*then $K_{\ell }=Z_{\ell }H_{\ell }^{-1}$ is a robust partially mode-dependent state-feedback control gain that assures that MJLS (6) is ESMS-CI in closed-loop with the SF control law (11).*

**Proof.**  In the proof, the dependence on α(k+1) is replaced by a superscript index + and α(k) is omitted to save space. For $A_{{cl}_{i}}^{\ell }=A_{i}+B_{i}K_{\ell },$ observe that (13) can be rewritten as $Q+XB+B^{\prime }X^{\prime }<0$ with $X={\left[\begin{array}{cc} H_{\ell } & 0 \end{array}\right]}^{\prime }$, $B=\left[\begin{array}{cc} -I & A_{{cl}_{i}}^{\ell }{}^{\prime }\Upsilon_{i} \end{array}\right]$, and $Q=\mathrm{Diag}\left(W_{i},\;W^{+}\right)$.

By choosing $B^{⊥}={\left[\begin{array}{cc} \Upsilon_{i}^{\prime }A_{{cl}_{i}}^{\ell } & I \end{array}\right]}^{\prime }$ as a base for the null space of B, and then multiplying (13) on the left by $B^{⊥}{}^{\prime }$ and on the right by $B^{⊥}$, one obtains $\Upsilon_{i}^{\prime }A_{{cl}_{i}}^{\ell }W_{i}A_{{cl}_{i}}^{\ell }{}^{\prime }\Upsilon_{i}-\mathrm{Diag}\left(\Upsilon_{i}\right)W^{+}<0$. Then, applying a Schur complement in the resulting inequality and pre- and post-multiplying by $\mathrm{Diag}\left(I,W_{i}^{-1}\right)$, one has

(14) $$\begin{array}{c} \left[\begin{array}{cc} I & 0\\ 0 & W_{i}^{-1} \end{array}\right]\left[\begin{array}{cc} -\mathrm{Diag}\left(\Upsilon_{i}\right)W^{+} & ⋆\\ W_{i}A_{{cl}_{i}}^{\ell }{}^{\prime }\Upsilon_{i} & -W_{i} \end{array}\right]\left[\begin{array}{cc} I & 0\\ 0 & W_{i}^{-1} \end{array}\right]<0, \end{array}$$

(15) $$\begin{array}{c} \Rightarrow \left[\begin{array}{cc} -\mathrm{Diag}\left(\Upsilon_{i}\right)W^{+} & ⋆\\ A_{{cl}_{i}}^{\ell }{}^{\prime }\Upsilon_{i} & -W_{i}^{-1} \end{array}\right]<0. \end{array}$$

By applying the Schur complement in (15) and making the following change of variable $W_{i}^{-1}=P_{i}$, one obtains (12), assuring the stability of the closed-loop MJLS. □

**Corollary** **1.** *Theorem 1 can be adapted to provide a stabilizing robust partially mode-dependent SOF control law (8) with an output matrix following Assumption 1 by imposing a diagonal structure on variable $H_{\ell }$ and a suitable structure (similar to the one presented in (10)) in variable $Z_{\ell }$, ∀ℓ∈Q.*

It is important to mention that, since the design conditions proposed in this section are parameter-dependent, they should be solved for all values of α(k)∈Λ (infinite dimensional problem), meaning that they are not computationally programmable. This issue can be surpassed by fixing the variables as homogeneous polynomials of fixed degrees and verifying the positivity (or negativity) of the resulting polynomial inequalities in terms of a finite set of LMIs automatically generated by the Robust LMI Parser (ROLMIP) toolbox [44].

## 6. Case Study

Consider the problem of control of a Vertical Take-Off and Landing (VTOL) helicopter whose state-space dynamic model in continuous-time is adapted from [45] and reproduced below

(16) $$\begin{array}{c} G_{c}=\left\{\begin{array}{cc} \dot{x}\left(t\right) & =A_{c}x\left(t\right)+B_{c}u\left(t\right)\\ y(t) & =C_{c}x\left(t\right) \end{array}\right.\\ A_{c}=\left[\begin{array}{cccc} -0.0366 & \;0.0271 & \;0.0188 & -0.4555\\ \;0.0482 & -1.0100 & \;0.0024 & -4.0208\\ \;0.1002 & \;0.3681 & -0.7070 & \;1.4200\\ \;0.0000 & \;0.0000 & \;1.0000 & \;0.0000 \end{array}\right],\\ B_{c}=\left[\begin{array}{c} \;0.4422\\ \;3.5446\\ -5.5200\\ \;0.0000 \end{array}\right],\;\;C_{c}=\left[\begin{array}{cccc} 0 & 0 & 0 & 1 \end{array}\right]. \end{array}$$

The states $x_{1}\left(t\right)$, $x_{2}\left(t\right)$, $x_{3}\left(t\right)$, $x_{4}\left(t\right)$ and the control input u(t), respectively, represent the horizontal and vertical velocities (knots), the pitch rate (degree/s), pitch angle (degrees), and the collective pitch control. Since the plant is remotely controlled through a wireless communication channel with a limited transmission rate, the control design is performed in discrete time. For this purpose, the matrices ($A_{c},B_{c},C_{c}$) from (16) are discretized using the zero order hold (‘zoh’) method in Matlab employing a fixed sampling period $T_{s}=0.1s$, resulting in discrete-time matrices (*A*,*B*,*C*). Then, a transfer function for the classic PID controller [18,19,20] (assuming ideal channel: no packet loss) is computed using the ‘pidTuner’ command from Matlab’s Control System Toolbox [46]:

$$PID\left(z\right)=\frac{-1.383z^{2}+2.567z-1.191}{0.05119z^{2}-0.002374z-0.04881}$$

which is equivalent to the following dynamic controller

(17) $$\begin{array}{c} G_{pid}=\left\{\begin{array}{cc} x_{c}\left(k+1\right) & =A_{pid}x_{c}\left(k\right)+B_{pid}y\left(k\right)\\ u(k) & =C_{pid}x_{c}\left(k\right)+D_{pid}y\left(k\right) \end{array}\right.\\ A_{pid}=\left[\begin{array}{cc} 0.0464 & 0.9535\\ 1 & 0 \end{array}\right],\;B_{pid}=\left[\begin{array}{c} 1\\ 0 \end{array}\right],\\ C_{pid}=\left[\begin{array}{cc} 48.8936 & -49.0271 \end{array}\right]\;D_{pid}=-27.0170. \end{array}$$

**Figure 5:**
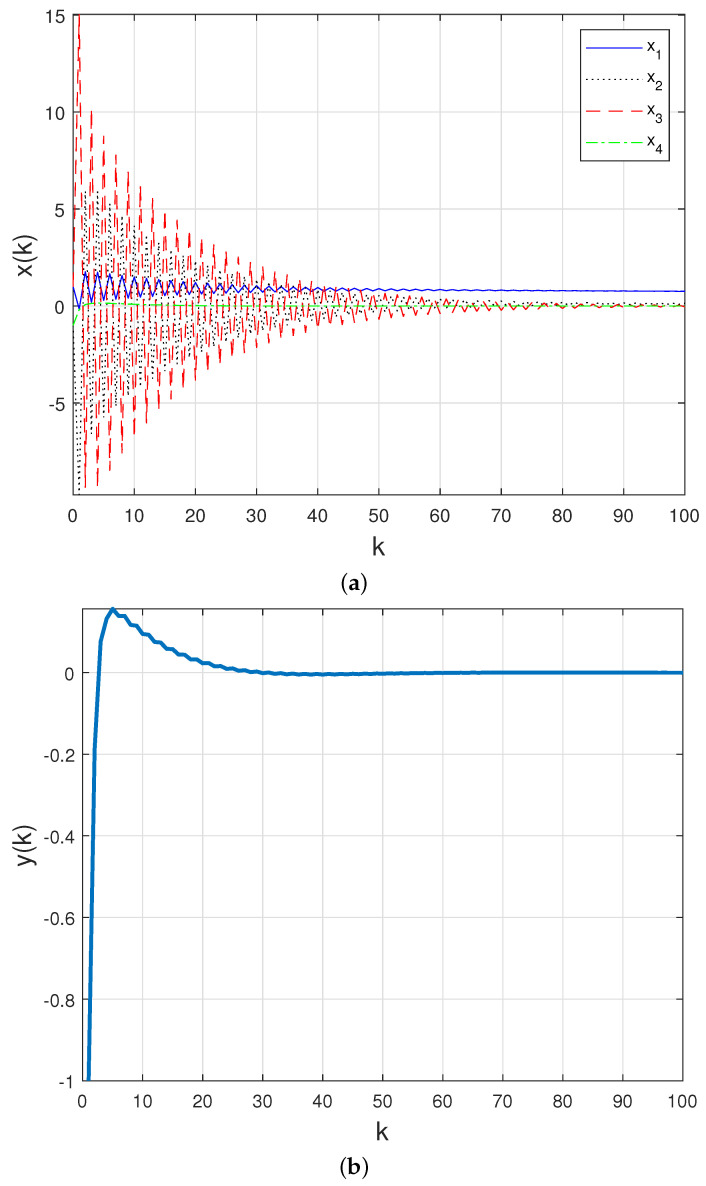
(**a**) Closed–loop states and (**b**) closed–loop measured output with PID controller (17) in a fully reliable network.

For the following initial conditions: $x\left(0\right)={\left[1,\;-1,\;1,\;-1\right]}^{\prime }$ and $x_{c}\left(0\right)={\left[0,\;0\right]}^{\prime }$, and assuming that the communication channel is fully reliable, the time-behavior of the closed-loop states (x(k)) and measured output (y(k)) presented in Figure 5a,b confirms that this controller is suitable when there is no packet loss on the network.

However, this system is controlled through a semi-reliable network with characteristics similar to the one emulated by the workbench described in Section 3. This means that the probability of the helicopter receiving a successful transmission of the control signal or the controller receiving a successful transmission of the sensor measurement can be considered as a time-varying parameter since it depends on the distance between the controller and the VTOL helicopter. When testing different attenuation levels between 1 dB and 25 dB in the wireless network workbench, it was noticed that PER increases monotonically with an increase in attenuation (representing, in this case, the distance between the nodes). This characteristic indicates that, at any distance between 0 m (1 dB: PER = 1%) and 310 m away (25 dB: PER = 11%), following the Gilbert-Elliot model presented in Section 3.1, the Markov chain can be represented by a transition probability matrix that is a convex combination of two vertices:

(18) $$\begin{array}{c} P\left(\alpha \left(k\right)\right)=\left[\begin{array}{cc} q(\alpha (k)) & \left(1-q(\alpha (k))\right)\\ 0.999 & 0.001 \end{array}\right]=\alpha_{1}\left(k\right)\left[\begin{array}{cc} 0.99 & 0.01\\ 0.999 & 0.001 \end{array}\right]+\alpha_{2}\left(k\right)\left[\begin{array}{cc} 0.89 & 0.11\\ 0.999 & 0.001 \end{array}\right] \end{array}$$

where 1−q(α(k)) corresponds to the packet error rate (see (5)), which varies from a lower bound ($\underline{PER}=0.01$, when the distance between the controller and the plant can be neglected) to an upper bound ($\bar{PER}=0.11$, when the distance between the controller and the plant matches the maximum range of the radio signal used for communication: 310 m). Note that, since the distance between the nodes does not remain constant, this means that both transition probability matrix P and, consequently, the parameter α are time-varying (depend explicitly on *k*). Additionally, as discussed in Section 3.1, the Markov model considers two operation modes: (i) successful transmission of the control and measured signals, (ii) failure. In this example, the packet loss of the control signal is modeled by the zero-input [47] approach, which is equivalent to clearing the elements of both the control and output matrices in the mode associated with transmission failure ($B_{2}=0$, $C_{2}=0$) and maintaining their actual values ($B_{1}=B$ and $C_{1}=C$ obtained from the discretization of (16)) in the operation mode that represents success. The dynamic matrix maintains its value in both modes $A_{1}=A_{2}=A$ obtained from the discretization of $A_{c}$ of (16).

**Figure 6:**
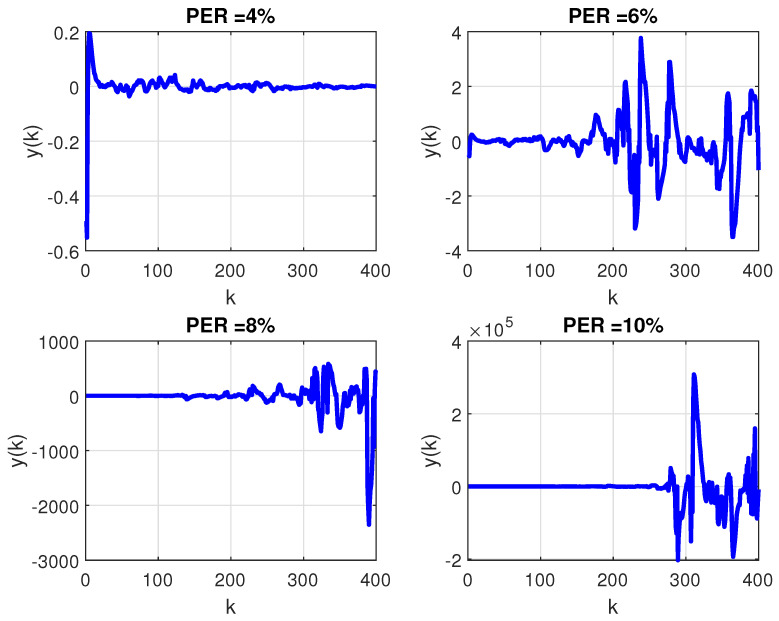
Mean of $10^{4}$ MC realizations of the Markov chain for closed–loop measured output with PID controller (17) through WSN with transition probability matrix given by (18) with constant PER equal to 4, 6, 8, 10% (clockwise, starting from top left frame).

**Figure 7:**
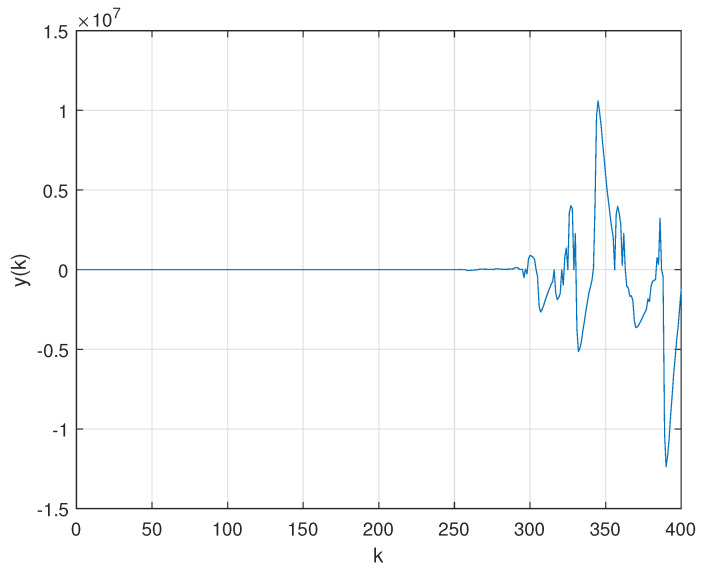
Mean of $10^{4}$ MC realizations of the Markov chain for closed–loop measured output with PID controller (17) through WSN with transition probability matrix given by (18) with a randomly time–varying PER.

In order to evaluate the robustness of PID control against the variation of the transition probability matrix, the mean of $M=10^{4}$ Monte Carlo (MC) realizations of the Markov chain were performed considering: firstly, a constant PER and, secondly, the extreme case of a randomly time-varying PER. The evolution of the closed-loop output considering the PID controller for the first case is shown in Figure 6, while the second case is illustrated by Figure 7. Note that, differently from what was reported in Figure 5, under the circumstance of packet loss, (whether constant or time-varying) the PID controller can no longer stabilize the plant.

Once a classic control design method proved ineffective in this NCS problem, a more suitable technique to design a controller robust to the variation of the transmission probabilities, given by Corollary 1, is employed next. First, it is assumed that the knowledge about the failure of the transmission is not available for feedback, then a mode-independent controller is synthesized by considering a single cluster containing both modes ($Q=\left\{1,2\right\}$) on Theorem 1.

Additionally, in order to provide an SOF controller, the following structure constraint is imposed to variable $Z=\left[\begin{array}{cccc} 0 & 0 & 0 & z_{4} \end{array}\right]$ in Theorem 1 since the discrete-time output matrix (*C*) is equal to the continuous-time one ($C_{c}$ from (16)). The SF control gain resulting from Theorem 1 is $K=\left[\begin{array}{cccc} 0 & 0 & 0 & 1.0959 \end{array}\right]$, meaning that the SOF control law obtained by Corollary 1 is

(19) u(k)=1.0959y(k).

**Figure 8:**
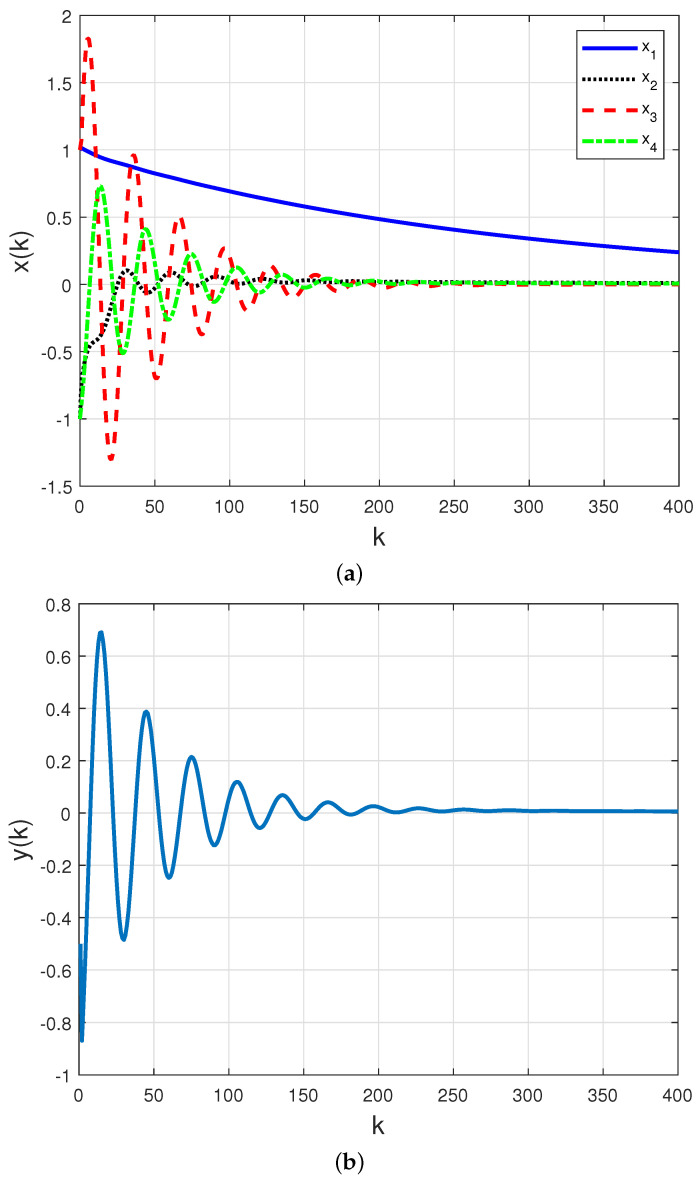
Mean of $10^{4}$ MC realizations of the Markov chain for (**a**) closed–loop states and (**b**) measured output with the SOF controller (19) obtained by Corollary 1 through WSN with transition probability matrix given by (18) with a randomly time–varying PER.

The time responses for $10^{4}$ MC realizations of the Markov chain for both closed-loop states x(k) and measured output y(k) are presented in Figure 8a,b considering a non-homogeneous Markov chain, governed by (18) with randomly time-varying PER.

In addition to stabilizing, the numerical complexity associated with the implementation of the SOF Markovian control (19) is another advantage when compared with the traditional PID controller (17). Notice that, while the computation of (19) consists of a product of scalar variables, the PID controller is required to solve the dynamic state–space equations of (17). Furthermore, the magnitude of the entries of matrices in (17) is approximately 50 times the magnitude of the Markovian controller (19), meaning that the use the PID controller requires a more robust actuator.

## 7. Conclusions and Future Work

The first contribution of this paper was the new approach to handle the problem of static output-feedback networked control design, considering that the wireless communication channel admits packet loss with a rate that can vary according with the distance between the nodes. The control design conditions were developed initially to provide theoretical assurances of closed-loop state-feedback stability for non-homogeneous MJLS, where the time-varying parameters affect the probabilities (and consequently, the Markov chain). However, an adaptation of the method to cope with SOF control problem was also presented, which is most widely explored in practical applications, since usually not all states are available for feedback.

The second contribution consisted of obtaining data related to the sequence of packets sent and received in a semi-reliable network by performing experiments on a wireless network workbench (previously developed and validated by the authors in real scenarios). Such data allowed us to find a Gilbert-Elliot model that describes the probability of packet loss in the communication channel. The transition probability associated with the communication channel model was employed in a practical motivated case study. The numerical experiments of the case study show that: (i) traditional control techniques such as PID cannot be applied to problems of networked control by semi-reliable communication channels since they do not guarantee the stability of the system when there is a possibility of packet loss, as shown in Figure 6 and Figure 7. On the other hand, the proposed technique based on the non-homogeneous Markov chain model is feasible and also ensures the stability of the closed-loop system, as shown in Figure 8; (ii) the numerical complexity associated with the implementation of a traditional PID controller (17) is much greater than the computational effort required to implement the static output feedback Markovian controller (19), requiring more calculations and better actuators, since the dimension and the magnitude of the controller are greater.

Although, in this paper, the wireless network workbench has been used only as a source of statistical data to obtain the Markov model for the wireless communication channel, as a future work, the authors intend to carry out an implementation of a similar control project on the workbench itself. The initial idea is to attach a microcontroller to each one of the network nodes so that the control and system dynamics are calculated online by the microcontrollers and the measurement and actuation signals are transmitted in real time by the emulated network. The third microcontroller that rules the actions of the digital signal attenuator will also be reprogrammed to implement an oscillatory behavior in order to emulate the variation of the distance between nodes. Other future investigation topics include: to consider different types of routing and models for the communication channel, and the study of energy efficiency and life cycle of the nodes considering large-scale WSNs, in which reliability of statistical data and the fluctuation of network factors, as well as the packet error rate (PER), become more critical.

## Figures and Tables

**Table 1:** Path loss exponent in various environments [38].

Environment	*n*
Free space	2
Urban area	2.7 to 3.5
Shadowed urban area	3 to 5
In building line of sight	1.6 to 1.8
Obstructed in building	4 to 6
Obstructed in factories	2 to 3

**Table 2:** Standard deviation values for distinct environments [38].

Environment	$\sigma_{\mathrm{dB}}$
Urban (distance <1 km)	3 to 4
Urban	8
Suburban	8
Indoor Small Office	4 to 6
Indoor Hot Spot	1.1 to 1.5
Outdoor to Indoor	7
Open Rural	6 to 8

**Table 3:** Workbench Results Against Reference Networks.

	No. of	Min.	Max.	Mean	Stand.
	Samples	RSSI	RSSI	RSSI	Dev.
Real test A	98,000	−67.00	−59.50	−62.78	1.13
Workbench	399	−64.50	−60.50	−62.21	0.62
Real test B	48,000	−83.00	−73.50	−78.05	1.24
Workbench	397	−84.50	−77.50	−80.88	1.2
Real test C	48,000	−70.00	−58.50	−63.47	0.62
Workbench	397	−65.00	−61.00	−63.01	0.38

**Table 4:** Workbench Results.

	Attenuation	No. of	Stand.	Success	PER
		Samples	Dev.		
Test 1	25	250	1.13	222	11%
Test 2	1	250	1.13	247	1%

**Table 5:** Example of time-evolution for a Markov chain $\theta_{k}$.

Packets sent	1	2	3	4	5	6	7	8	9	10
Packets received	1	-	3	4	-	6	7	8	9	10
$\theta_{k}$ (success = 1, failure = 2)	1	2	1	1	2	1	1	1	1	1

## Data Availability

Not applicable.

## Abbreviations

The following abbreviations are used in this manuscript:

WSN	Wireless Sensor Network
PLR	Packet Loss Rate
PER	Packet Error Rate
MJLS	Markov Jump Linear System
PID	Proportional-Integral-Derivative
VTOL	Vertical Take-Off and Landing
NCS	Networked Control System
OSI	Open Systems Interconnection
SMS	Second Moment Stability
MSS	Mean Square Stability
LMI	Linear Matrix Inequality
SOF	Static Output-Feedback
SF	State-Feedback
ESMS-CI	Exponential Stability in the Mean Square sense with Conditioning type I
ROLMIP	Robust LMI Parser
ISM	Industrial, Scientific, and Medical
PC	Personal Computer
USB	Universal Serial Bus
RF	Radio Frequency
RSSI	Received Signal Strength Indicator
zoh	Zero Order Hold
MC	Monte Carlo
